# Extracellular Vesicles Mediate Immune Responses to Tissue-Associated Self-Antigens: Role in Solid Organ Transplantations

**DOI:** 10.3389/fimmu.2022.861583

**Published:** 2022-04-27

**Authors:** Ranjithkumar Ravichandran, Sandhya Bansal, Mohammad Rahman, Angara Sureshbabu, Narendra Sankpal, Timothy Fleming, Ankit Bharat, Thalachallour Mohanakumar

**Affiliations:** ^1^ Norton Thoracic Institute, St. Joseph’s Hospital and Medical Center, Phoenix, AZ, United States; ^2^ Department of Surgery-Thoracic, Northwestern University, Chicago, IL, United States

**Keywords:** extracellular vesicles, transplantation, immune responses, tissue-associated self-antigens, antibodies

## Abstract

Transplantation is a treatment option for patients diagnosed with end-stage organ diseases; however, long-term graft survival is affected by rejection of the transplanted organ by immune and nonimmune responses. Several studies have demonstrated that both acute and chronic rejection can occur after transplantation of kidney, heart, and lungs. A strong correlation has been reported between *de novo* synthesis of donor-specific antibodies (HLA-DSAs) and development of both acute and chronic rejection; however, some transplant recipients with chronic rejection do not have detectable HLA-DSAs. Studies of sera from such patients demonstrate that immune responses to tissue-associated antigens (TaAgs) may also play an important role in the development of chronic rejection, either alone or in combination with HLA-DSAs. The synergistic effect between HLA-DSAs and antibodies to TaAgs is being established, but the underlying mechanism is yet to be defined. We hypothesize that HLA-DSAs damage the transplanted donor organ resulting in stress and leading to the release of extracellular vesicles, which contribute to chronic rejection. These vesicles express both donor human leukocyte antigen (HLA) and non-HLA TaAgs, which can activate antigen-presenting cells and lead to immune responses and development of antibodies to both donor HLA and non-HLA tissue-associated Ags. Extracellular vesicles (EVs) are released by cells under many circumstances due to both physiological and pathological conditions. Primarily employing clinical specimens obtained from human lung transplant recipients undergoing acute or chronic rejection, our group has demonstrated that circulating extracellular vesicles display both mismatched donor HLA molecules and lung-associated Ags (collagen-V and K-alpha 1 tubulin). This review focuses on recent studies demonstrating an important role of antibodies to tissue-associated Ags in the rejection of transplanted organs, particularly chronic rejection. We will also discuss the important role of extracellular vesicles released from transplanted organs in cross-talk between alloimmunity and autoimmunity to tissue-associated Ags after solid organ transplantation.

## Introduction

Emerging research shows that small extracellular vesicles (sEVs) are potential biomarkers and immune mediators in the field of transplantation (1–3). After lung transplantation (LTx), sEVs identified in the circulation and locally in the bronchoalveolar lavage fluid had distinct RNA profiles under normal and inflammatory conditions (4, 5). Our reports demonstrate the presence of mismatched donor human leukocyte antigens (HLAs) and lung associated-antigens on sEVs surfaces after LTx, suggesting that sEVs with lung TaAgs can be a biomarker for monitoring allograft rejection (6). SEVs derived from donor dendritic cells have been demonstrated to promote allograft-targeting immune responses by transferring immune-activating signals and donor HLA molecules (7). Studies have shown that the development of *de novo* antibodies (Abs) to mismatched donor-HLA is associated with chronic rejection after LTx, which is clinically diagnosed as bronchiolitis obliterans syndrome (BOS) (8, 9). Evidence also suggests that anti-donor responses leading to rejection are often due to *de novo* donor-specific antibodies (HLA-DSAs) and donor HLA reactive immune T cells (cellular rejection) (10). Recent evidence clearly demonstrates that Abs against donor HLA present either before transplantation or developed *de novo* after transplantation are strongly associated with antibody-mediated rejection (AMR), and repeated episodes of AMR are an important risk factor for chronic lung allograft dysfunction (CLAD) (11, 12). We have demonstrated that the development of Abs to TaAgs increases the risk for the development of HLA-DSAs and vice versa, indicating crosstalk between allo- and auto-immunity, both of which are implicated in the development of BOS (13). Abs to HLAs combined with a loss of the T regulatory cell population is also implicated in chronic rejection (14, 15). Several risk factors have been identified for chronic rejection, including donor organ ischemia, HLA mismatches, development of *de novo* HLA-DSAs, recurrent/refractory acute rejections, and viral infections (16, 17). We postulate that any of these risk factors can lead to inflammation and tissue remodeling, which facilitates the induction and release of extracellular vesicles (EVs), leading to immune responses against donor alloantigens and TaAgs and the development of allo- and auto-immunity. Although sEVs may have many relevant biological functions, including the induction of rejection and/or tolerance, data supporting the contribution of sEVs to these processes are currently limited. Therefore, the mechanisms by which sEVs regulate immune responses need further investigation.

## Immune Responses Against HLA and Non-HLA TaAgs and Allograft Rejection

Immune responses are recognized immediately after organ transplantation due to ischemia and reperfusion injury of the transplanted organ (18–21). Recent studies demonstrate a strong correlation between ischemia-reperfusion injury and *de novo* HLA-DSAs (22), thus increasing the risk for development of chronic rejection after human LTx (23–25), and many reports strongly support the concept that *de novo* HLA-DSAs after transplantation can lead to allograft dysfunction, including AMR (23, 26–28). It is also clear that *de novo* HLA-DSAs are an important player in allograft dysfunction during cellular rejection as many transplant recipients with cellular rejection have evidence of both T cells mediated immune response and Abs specific to mismatched HLA (29–31). *De novo* HLA-DSAs are an established biomarker for predicting AMR (32–35) and also play a significant role in the immune-pathogenesis of chronic rejection (36). Earlier studies have demonstrated that even a transient presence of *de novo* HLA-DSAs can be an important risk factor for the development of chronic rejection, indicating that persistent HLA-DSAs may not be necessary (37, 38).

Employing sera from LTx recipients diagnosed with BOS but without detectable HLA-DSAs in the circulation, we defined immune responses to lung tissue–associated Ags expressed on airway epithelial cells and their possible role in the development of BOS (39). Further, serial analysis of antibody development after LTx demonstrated that HLA-DSAs can induce immune responses to lung TaAgs, which may lead to the pathogenesis of chronic rejection (13). From these studies, we concluded that HLA-DSAs may induce immune responses to TaAgs, which either alone or in combination of both increases the risk of BOS. Significantly, primary graft dysfunction (PGD) is also a well-known risk factor for the development of BOS (40–42). Inflammation and tissue remodeling in the recipient and the surgical stress during transplantation can lead to the expression of sequestrated antigenic epitopes of TaAgs present in the lungs, and pre-existing immune responses to TaAgs can lead to PGD (43). Therefore, it is important to determine the role of pre-existing immune response to TaAgs after LTx so that rejection can be avoided.

Several well-studied non-HLA antigens may play a role in immune responses following organ transplantation. Immune responses against collagen V (Col-V) and K-alpha 1 tubulin (Kα1T) have been shown to play an important role in eliciting both cellular and humoral immune responses leading to lung allograft rejection (44, 45). Furthermore, Abs against cardiac myosin (MYO) and vimentin (VIM) have been reported to be present during cardiac allograft rejection (46, 47). After human kidney transplantation, Abs against perlecan (48), Col-IV, and fibronectin (FN) have been associated with rejection (49). In addition, immune responses against major histocompatibility complex (MHC) class I-related chain A (MICA) molecules have been identified in patients undergoing lung, kidney, and heart allograft rejection (50–52). These Abs to non-HLA antigens often precede the diagnosis of chronic rejection in lung, heart, and kidney recipients, suggesting a causal relationship (50–52). Recently, the International Society of Heart and Lung Transplantation guidelines proposed AMR as a distinct clinicopathological entity characterized by the presence of allograft dysfunction in concert with histological findings of capillary injury, positive immunofluorescence for C4d in lung tissue biopsies, and detection of circulating HLA-DSAs (11, 53). Accumulating evidence now suggests that early post-transplant events promote the development of inflammatory processes which subsequently lead to the development of *de novo* HLA-DSAs, increasing the risk for rejection of the transplanted organ (9, 27, 35). We demonstrated the role of HLA-DSAs to mismatched HLA molecules and Abs against two important lungs TaAgs (Col-V, Kα1T) in LTx recipients diagnosed with BOS (54–56). The presence of HLA-DSAs during AMR has also been associated with the development of Abs to Col-V and Kα1T in LTx recipients (55).

## Tissue Associated Ags: Role in Lung Allograft Rejection

Col-V is a collagen intercalated within fibrils of type I collagen present in lung tissue. Yoshida et al. have demonstrated that Col-V is exposed and also released in bronchoalveolar lavage fluid due to ischemia-reperfusion injury during LTx (57). Iwata et al. have shown that graft injury mediated by HLA-DSAs can also modify Col-V and release Col-V fragments that serve as a major target in the transplanted organ for the development of Abs against TaAgs (58). Our laboratory analyzed pre-transplant sera from 317 LTx recipients between 2000 and 2011 with a diagnosis of chronic obstructive pulmonary disease, idiopathic pulmonary fibrosis, cystic fibrosis, or other end-stage lung diseases for Abs to Col-V and demonstrated that patients with idiopathic pulmonary fibrosis and cystic fibrosis had the highest prevalence of Abs to lung TaAgs. The Abs to lung TaAgs increased the risk for development of HLA-DSAs, PGD, and BOS (59). Tiriveedhi et al. demonstrated that Col-V epitopes shift from both α1 and α2 to only α1during BOS; this shift in immunodominant epitopes is correlated with a decreased expression of IL-10 and increased expression of IFN-γ (60). Col-V-specific Th17 cells and monocyte/macrophage accessory cells have been shown to cause progressive airway obliteration (61). *De novo* immunity to Col-V resulting in BOS was associated with donor-lung HLA-DR15+, suggesting that donor-derived HLA-DR15 presents novel self-peptides to recipient T cells (62).

We also demonstrated that administration of Abs to lung TaAgs (Col-V, Kα1T) in animal models of LTx resulted in antigenic epitope spreading between lung TaAgs and allo-MHC molecules leading to marked lung graft cellular infiltration, bronchiolar fibrosis, and loss of tolerance (63). Passive administration of Abs to Kα1T also led to cellular and humoral immune responses to Col-V, demonstrating a spreading of immune responses leading to the development of fibrosis in the transplanted organ and signifying the importance of immune responses to TaAgs in the pathogenesis of chronic rejection (45).

Studies have shown that tolerance to a lung allograft can be induced when CD4+ Col-V-specific regulatory T cells, which can downregulate Th17-mediated acute rejection, are administered after LTx (64). T regulatory cells that mediate Col-V-induced tolerance to lung allografts are phenotypically CD4^+^CD45RC high, lacking Smad7, and have not been associated with TGF-β-mediated signaling (65). We also investigated the role of B cells and their antigen-presenting properties in the induction of obliterative airway disease model where Abs to MHC class I were administered in B cell ^−/−^ and wild-type mice. Incidence of Kα1T- and Col-V-specific IL-17 cells significantly decreased in B^−/−^ mice, and Abs against TaAgs and germinal center formation did not develop in B^−/−^ mice (66). Our study has also described that induction of a transcription factor, zinc finger, and BTB domain-containing protein 7a (Zbtb7a) in alveolar macrophages were critical regulators of the inflammatory circuit associated with development of Abs to TaAgs in an obliterative airway disease model (67).

Kα1T is an epithelial gap junction-associated protein expressed also in the lungs (36). In a clinical study of adult LTx recipients (N=142), we demonstrated that the presence of Abs to Kα1T pre-transplant led to an increase in proinflammatory cytokines IL-1 (2.1 fold increase), IL-2 (3.0 fold increase), IL-12 (2.5 fold increase), and IL-15 (3.0 fold increase) and chemokines IP-10 (3.9 fold increase) and MCP-1 (3.1 fold increase) and increased the risk of PGD and BOS (68).

Budding et al. demonstrated the presence of anti-ETAR and anti-AT1R auto-Abs in both pre- and post-transplant sera from 43 LTx patients transplanted due to chronic obstructive pulmonary disease, cystic fibrosis, or interstitial lung disease (69). Pre- and post-transplant sera from 162 LTx recipients at three centers between 2011 and 2013 were tested for Abs to AT1R and ETAR. Recipients with strong/intermediate binding of Abs to AT1R and ETAR had increased AMR onset and development of *de novo* HLA-DSA (70). After organ transplantation, development of Abs to AT1R and ETAR is likely due to damage to the endothelium of the host or transplanted organ followed by shedding of the extracellular particles of the receptors (71).

## TaAgs in Cardiac Allograft Rejection

Abs to tissue-associated antigens have been validated as important mediators of rejection, allograft dysfunction, and cardiac allograft vasculopathy (CAV) after heart transplantation (72, 73). In heart transplant recipients, increased levels of AT1R have been associated with AMR, cell-mediated rejection, and early onset of micro vasculopathy at 1-year post-transplant (74). A recent study has demonstrated an association of VIM Ab with other non-HLA Abs in heart transplant recipients even when the patient is treated for AMR (75). Analysis using a non-HLA antigen multiplex panel has also documented, and validated, an important role for non-HLA Abs in heart transplantation (76).

An earlier study demonstrated allogeneic heart transplantation in mice resulted in a breakdown of immune tolerance to cardiac MYO, and pre-transplant sensitization of recipient mice with cardiac MYO caused a marked acceleration of rejection of allogeneic heart grafts. This strongly suggests that allogeneic transplant-induced autoimmunity to cardiac MYO contributes to cardiac graft rejection (77). Humoral autoimmune responses against cardiac MYO were associated with dilated cardiomyopathy and increased the frequency and severity of acute rejection after heart transplantation (78). Further studies that evaluated Abs to cardiac MYO in pre- and post-transplant sera from 41 adult cardiac allograft recipients suggested a pathogenic role for anti-MYO Abs in both acute and CAV-related heart transplant rejection (46, 79).

Earlier studies in nonhuman primates also demonstrated the role of humoral immunity to VIM and its association with cardiac allograft injury (80). Mahesh B, et al. (81, 82) demonstrated that autoimmune responses to VIM in conjunction with alloimmune responses accelerate CAV progression in murine cardiac allografts.

## Renal SAgs: Role in Kidney Transplantation

Tissue-associated Ags are cryptic epitopes that can serve as targets for the development of autoimmunity when exposed (83). Studies have demonstrated that Abs against kidney TaAgs contribute to the process of acute AMR after kidney transplantation (83, 84) and to the development of transplant glomerulopathy (49). Well-characterized renal TaAgs include AGT1R perlecan, Col-IV, and FN. Perlecan, a proteoglycan embedded within the vascular basement membrane, when degraded by metalloproteinases or cathepsin-L, releases endorepellin and a truncated C-terminal fragment harboring a laminin G motif (LG3) (85). Increased urinary levels of LG3 have also been observed in kidney transplant recipients with chronic rejection (86, 87). Endothelial apoptosis is associated with acute and chronic vascular rejection of solid allografts (88). Apoptotic endothelial cells release LG3, a regulator of obliterative vascular remodeling during rejection, which can lead to development of autoimmunity (86). Yang et al. demonstrated that the presence of pre-transplant LG3 was associated with reduced allograft function at 1 year in kidney transplant recipients; severity of renal injury at the time of transplantation had a negative impact on long-term outcomes (89). Recent studies have also demonstrated that Abs against perlecan can cause acute humoral rejection in pre-sensitized patients regardless of development of Abs to HLA after kidney transplantation (90).

Col-IV is a major constituent of glomerular basement membranes (91, 92). Studies have demonstrated that Col-IV is upregulated in acutely deteriorated renal allografts, and differential staining of Col-IV in glomerular basement membranes and the interstitium may help to diagnose chronic transplant nephropathy (93).

BK virus–associated nephropathy (94) and persistent BK viremia (defined as lasting >140 days) were also associated with *de novo* development of Abs against mismatched donor HLA antigens (HLA-DSA) (95). We demonstrated polyomavirus reactivation was associated with early *de novo* development of Abs to the kidney-associated Ag FN; the immune responses to FN along with Col-IV were associated with an increased risk of acute rejection (96). Dragun et al. first demonstrated the role of Abs to AT1R and its association in renal transplant recipients with acute vascular rejection with refractory hypertension (97). Abs to AT1R that bind to G-protein–coupled receptors can induce stress in endothelial cells *via* activation of distinct intracellular cell-signaling pathways (98). Several studies have associated pre- and post-transplant AT1R and ETAR Abs with AMR and adverse late graft outcomes in kidney transplantation (99–101).

## EVs: Role for Eliciting Immune Responses to TaAgs

**Figure 1:**
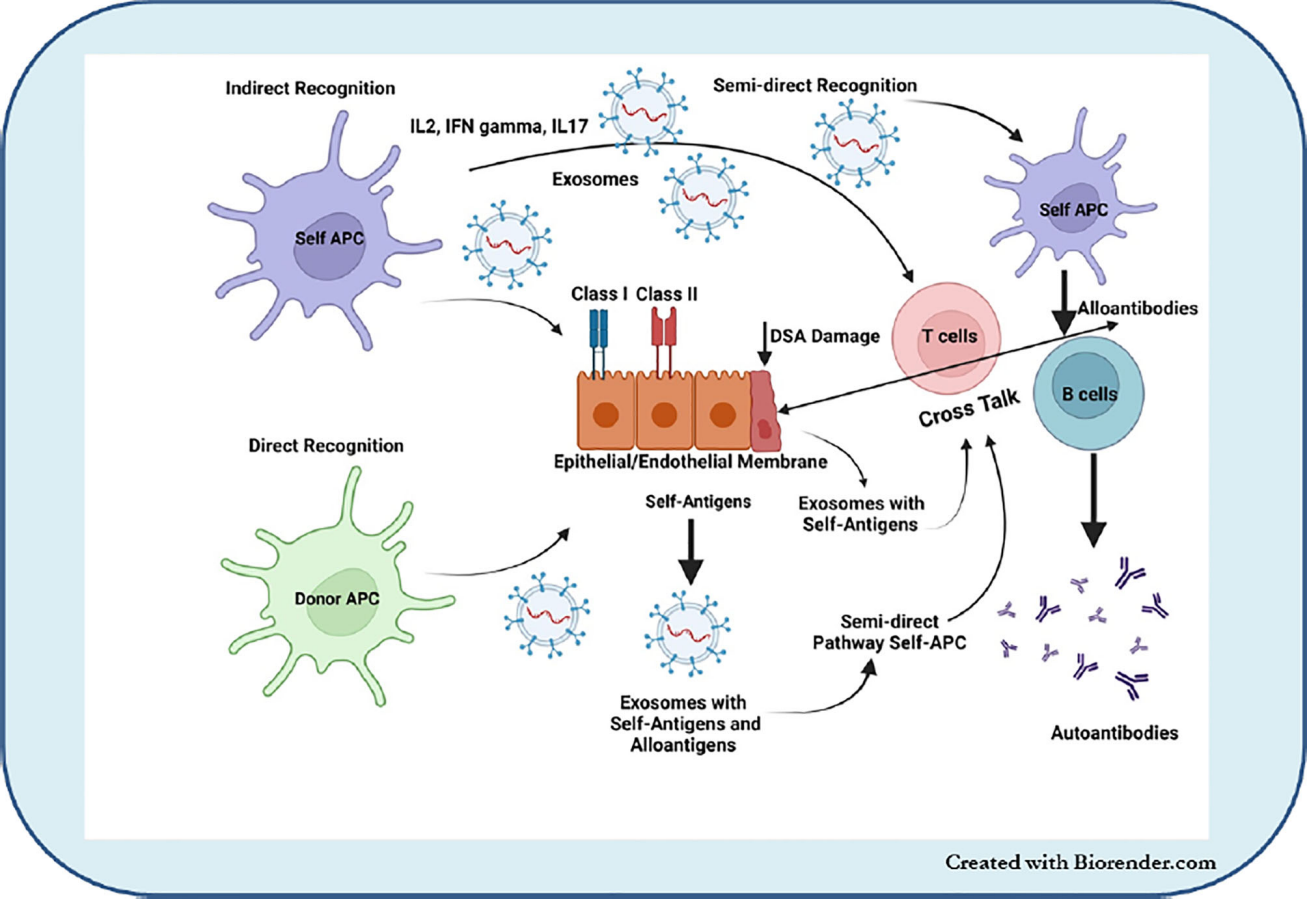
EVs interact with cells *via* numerous ligand–receptor interactions and sEVs can activate not only direct and indirect pathways of antigen presentation but also *via* the *semidirect pathway* in which T cell activation occurs *via* donor-derived sEVs.

Our group, including others, demonstrated that the development of Abs to mismatched donor HLA as well as immune responses to lung TaAgs are strongly associated with the development of BOS (55, 102, 103). Studies have shown that lung injury due to PGD, acute rejection, and respiratory viral infections, known risk factors for the development of CLAD, also induce circulatory sEVs with lung TaAgs (6, 104). sEVs (68, 105–107). Based on these results, we propose that stress to the transplanted organ either by PGD, respiratory viral infections, rejection episodes, or HLA-DSAs can release circulating sEVs with lung TaAgs. Persistence of these sEVs in the circulation can lead to continued immune activation and the development of allo- and auto-immunity, which can increase the risk of BOS. EVs interact with cells *via* numerous ligand–receptor interactions, and sEVs can activate not only *via* direct and indirect pathways of antigen presentation but also *via* the semidirect pathway, in which T cell activation occurs *via* donor-derived sEVs (**Figure 1**) (108, 109).

EVs, or sEVs, are released by all cells, and EVs are heterogeneous in size and composition. EVs are classified by size: microvesicles (100–1000 nm in diameter), apoptotic bodies (>50-5000 nm in diameter), and sEVs (50–150 nm in diameter) (110, 111). All the secreted vesicles carry proteins/peptides, lipids, nucleic acids (DNA, RNA), and small metabolites; the composition and functional impact of vesicles depend on their cellular origin (112). The composition of sEVs is critical as they can be a potential biomarker for function in biological processes. In addition, there are certain markers, apart from size, are enriched in sEVs than other vesicles (e.g., TSG101, syntenin, tetraspanins, CD9, CD63, and CD81) (113, 114). Focus here is to discuss the role and importance of sEVs in the development of immune responses to TaAgs (autoimmunity) in lung allograft and other solid organ transplant recipients.

**Figure 2:**
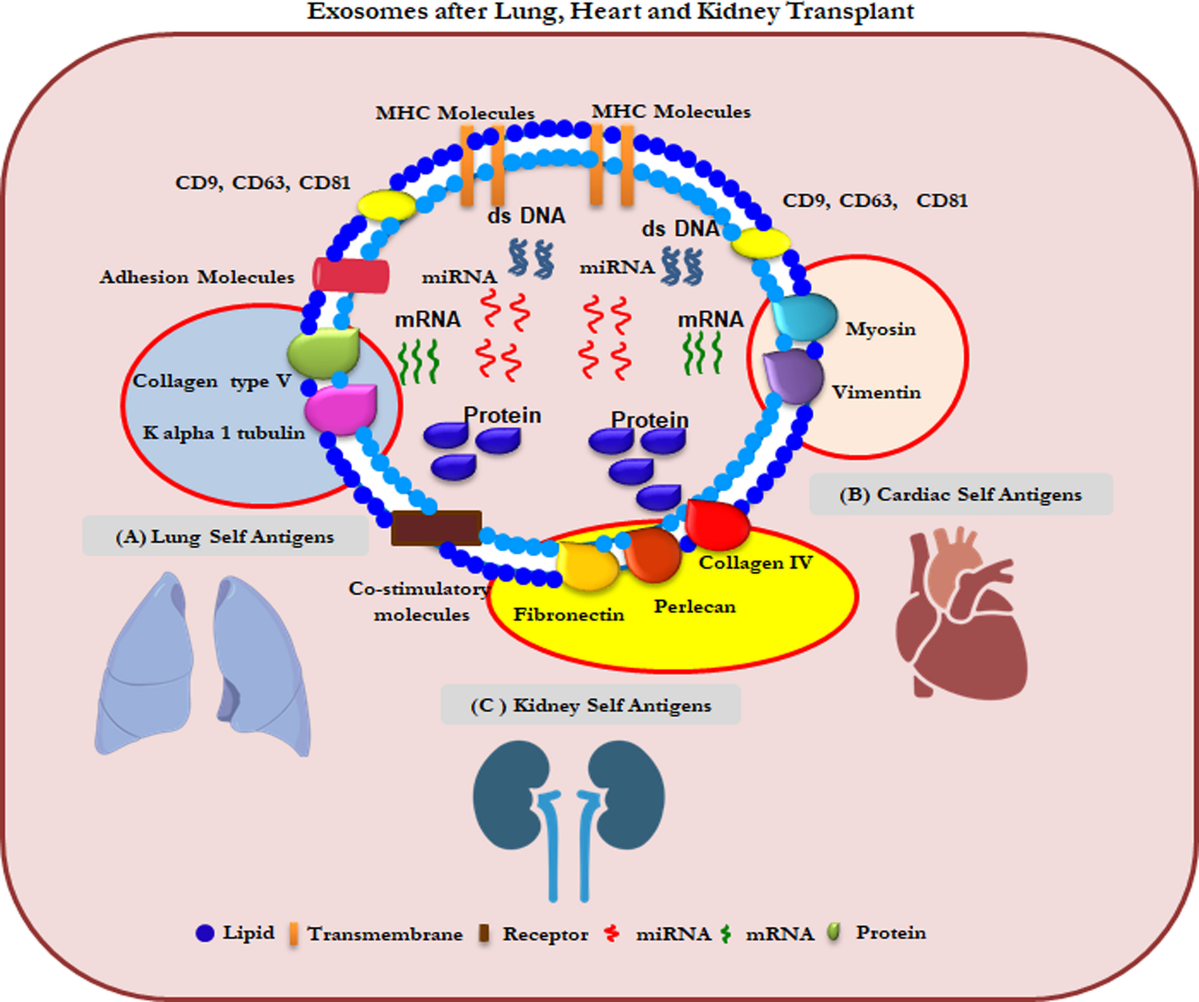
Composition of sEVs from lungs, heart and kidney transplant patients. **(A)** Col-V and Kα1T TAgs on lung specific sEVs **(B)** MYO and VIM TAgs on heart specific sEVs **(C)** Perlecan and Fibronectin TAgs on kidney specific sEVs.

SEVs are involved in several biological and immune processes (115). Our group has recently validated the use of circulating sEVs with lung TaAgs as a potential biomarker in the early diagnosis of BOS after LTx (116). In addition, different clinical conditions after LTx that increase the risk for CLAD, e.g., PGD, AR, HLA-DSAs, and respiratory viral infections, can induce circulating sEVs with lung TaAgs (117). Increasing evidence supports a possible role of sEVs in the pathogenesis of various human diseases (108, 118–120). We have reported that sEVs isolated from LTx recipients contain increased levels of the lung TaAgs (Col-V, Kα1T) during acute and chronic rejection (6). Preliminary studies have also shown association of sEV’s containing cardiac TaAgs (VIM and MYO) in syngeneic cardiac transplant rejection induced by antibodies to cardiac myosin rejection (121). Our preliminary studies also demonstrated sEV’s containing kidney antigens (FN and Col-IV) in kidney transplant recipients was associated with transplant glomerulopathy and interstitial fibrosis and tubular atrophy (IFTA) (unpublished data) (**Figure 2**).

## EVs in Eliciting Immune Responses

The development of alloimmunity and Abs to TaAgs (autoimmunity) after transplantation is likely due to the induction and release of sEVs from the transplanted organ under varying conditions resulting in stress to the allograft. We and others have demonstrated that circulating sEVs released from the transplanted organ indeed play an important part in eliciting immune responses leading to rejection (7, 109, 122, 123). The presence of mismatched donor HLA and lung TaAgs (Col-V, Kα1T) in sEVs isolated from LTx recipients diagnosed with rejection demonstrates that the sEVs originated from the transplanted organ (6, 104). In a murine model of chronic lung allograft rejection (124) in which a single lung from B6D2F1 (H2^b/d^) mice was orthotopically transplanted into DBA/2(H2^d^) recipients, sEVs containing mismatched donor MHC (H2b) and lung TaAgs (Col-V, Kα1T) were released (days 14 and 28), and Abs to donor MHC developed, a known risk factor for pathogenesis of CLAD (12, 27). Furthermore, passive administration of these sEVs to DBA/2 recipient mice led to the development of Abs to donor MHC (H2^b^) and Abs to lung TaAgs, signifying that sEVs stimulate immune responses that lead to rejection (125). SEVs also contained MHC class II molecules; costimulatory molecules CD40, CD80, and CD86; and transcription factors class II MHC trans-activator, NF-kB, hypoxia inducible factor-1α, IL-1R–associated kinase 1, MyD88, and 20S proteasome (123). These molecules often increase in sEVs in LTx recipients with BOS in comparison to stable LTx recipients (123). We have also demonstrated that sEVs isolated from LTx recipients with BOS are immunogenic *in vivo* since immunization of mice with isolated sEVs resulted in humoral and cellular immune responses to lung TaAgs (6).

In a recent study, we demonstrated that sEVs from LTx recipients with BOS also contained NK cell markers (CD56, NKG2D) and cytotoxic molecules (perforin, FasL) (126) and, therefore, can play an important role in antiviral and antitumor immune surveillance (127). In the chronic murine LTx rejection model discussed above, we demonstrated that circulating sEVs are also derived from NK cells since isolated sEVs contain NK cell–associated molecules (NKp46, CD56, NKG2D) and cytotoxic molecules (perforin, FasL) (125). Further, NK cell depletion significantly reduced fibrosis in transplanted lung tissue, suggesting a role for NK cells in the pathogenesis of chronic rejection in this murine model of LTx rejection (125).

Recent studies demonstrated that the two phenotypes of CLAD (BOS and restrictive allograft syndrome) can be differentiated by levels of specific proteins in circulating sEVs (128). SEVs from patients with the restrictive allograft syndrome phenotype carry higher amounts of costimulatory marker CIITA, transcription factor NFkB, lung TaAg Kα1T, and class II HLA-DQ and DR molecules and 20S proteasome.

A recent study demonstrated that EVs derived from vascular injury, ApoExo, as novel inducers of tertiary lymphoid structure (TLS) formation by attracting IL‐17‐producing gamma delta T cells to the allograft and ApoEXo proteasome activity was the important mediator of TLS formation (129). The formation of TLS in kidney allografts has been associated with severe and/or chronic forms of rejection. Earlier studies have demonstrated a stepwise breakdown of B-cell tolerance and formation auto antibodies during chronic rejection (130). The mechanism that occurs within the kidney graft during chronic rejection is due to changes in intragraft microenvironment which can be mediated by ApoExo released by local tissue injury (129, 131). A recent study demonstrating donor EVs were transported across the sub capsular sinus macrophages, and donor MHC molecules on the EVs were recognized by alloreactive B cells (132). This triggered B cell activation and DSA production, suggesting the EVs can activate immune response by semi direct pathway and does not always require donor leukocytes to directly interact for B cell allosensitization.

## Tumor Suppressor Gene Liver Kinase B1 (LKB1) and Lung Allograft Rejection

LKB1, also known as serine/threonine protein kinase 11, is a tumor suppressor gene (133, 134). By direct phosphorylation, LKB1 activates a family of 14 kinases related to the AMP-activated protein kinase (AMPK) (135–137). Most functions of LKB1 are attributed to its ability to activate AMPK, a central conserved regulator of metabolism and cell growth (138). AMPK regulates factors involved in cell metabolism, proliferation, survival, migration, and invasion (139–142). It has been demonstrated that loss of LKB1 is a biomarker for more aggressive biology in KRAS-mutant lung adenocarcinoma (6, 143). Studies have also demonstrated that expression of LKB1 inhibits epithelial-mesenchymal transition, tissue fibrosis, and malignant transformation (144, 145).

Recently, we demonstrated significantly reduced abundance of LKB1 in both mRNA/protein level in circulating sEVs isolated from LTx recipients diagnosed with BOS compared to sEVs isolated from stable LTx recipients (146). Our study also demonstrated novel findings indicating that sEV-mediated downregulation of a tumor suppressor gene, LKB1, in primary epithelial cells may also play an important role in chronic rejection after LTx by inducing epithelial-mesenchymal transition by upregulating VIM and α-SMA, leading to the pathogenesis of BOS (146).

## sEVs With Tissue-Associated Ags as a Biomarker for CLAD

A recent report by our group demonstrated that circulating sEVs isolated from plasma and bronchoalveolar lavage fluid from LTx recipients with BOS have donor HLA and significantly increased levels of lung TaAgs (Kα1T, Col-V) (6, 61). Based on this finding, we analyzed plasma samples collected from LTx recipients 6 and 12 months before the clinical diagnosis of BOS from two different LTx centers. Increased levels of lung TaAgs were detected in sEVs isolated from these samples 12 months before the clinical diagnosis of BOS, indicating that circulating sEVs with lung TaAgs can be a viable noninvasive biomarker for identifying patients at risk for developing CLAD (116). The determination of Col-V or Kα1T in circulating sEVs by western blot followed by semi-quantitation provides a higher positive predictive value with excellent sensitivity and specificity. During this study, the validation cohorts demonstrated that levels of sEVs containing Col-V had a sensitivity of 70% and a specificity of 80% at 6 months and a sensitivity of 60% and a specificity of 75% at 12 months before clinical diagnosis of BOS. For the lung TaAg, Kα1T, sensitivity was 60% and specificity was 80% at 6 months and sensitivity was 65% and specificity was 80% at 12 months. Early detection of patients at risk for developing chronic rejection provides an opportunity to develop strategies to prevent or intervene before the onset of irreversible damage to the transplanted organ.

We also found LKB1 expression was downregulated in circulating sEVs isolated from LTx recipients with BOS 6 months before the clinical diagnosis. Recently, it has been reported that aldehyde/sulfate latex beads can bind to sEVs and bead-sEVs can be detected by flow cytometry (147). Using this method, we demonstrated that LKB1 levels were significantly lower in sEVs isolated from LTx recipients 6 months before clinical diagnosis of BOS than in sEVs isolated from stable LTx recipients (146).

## Viral Antigens in Circulating sEVs

LTx recipients are prone to respiratory viral infections (respiratory syncytial virus, rhino, cytomegalovirus, and corona). Respiratory viral infections have been shown to increase the risk of CLAD, but the mechanisms remain largely unknown. One of the reports demonstrated that respiratory viral infections after LTx induce circulating sEVs that contain lung-associated Ags and viral antigens (117), and the viral antigens are on the surface of the isolated sEVs. These sEVs also induced immune responses to TaAgs when mice were immunized with isolated sEVs (123).

sEVs isolated from LTx recipients with respiratory viral infections also contained nucleic acid sequences of DNA and RNA viruses. These sequences in sEVs can induce innate immune signaling, cellular stress, and epithelial-mesenchymal transition both *in vitro* and *in vivo* through cGAS/STING and RIG1 pathways (148).

**Figure 3:**
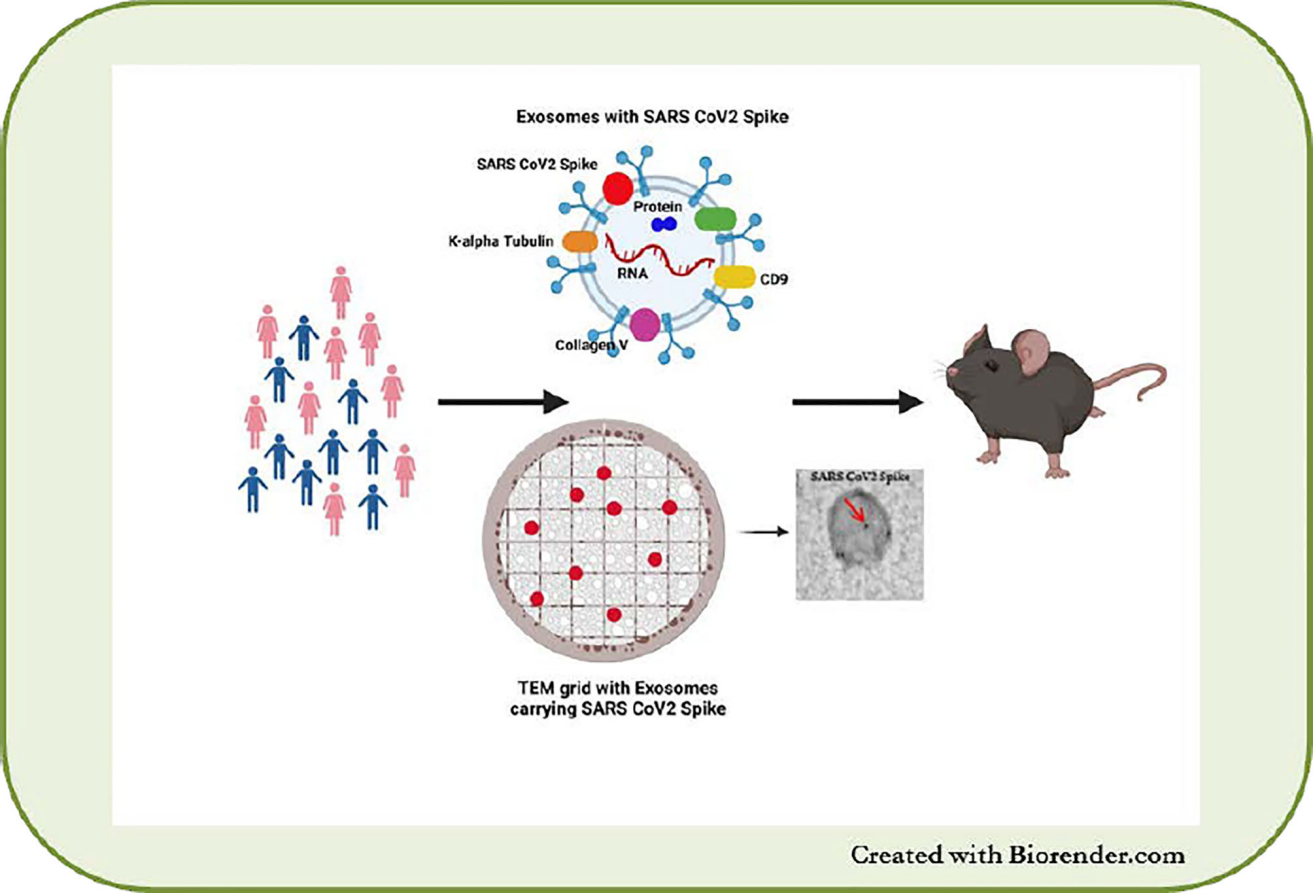
sEVs from SARS-CoV-2 infected and vaccinated patients. Presence of SARS-CoV-2 spike protein on sEVs and immunization of mice with the sEVs leads to development of SARS-CoV-2 antibodies in mice.

Recently, we demonstrated the physiological role of sEVs in eliciting immune responses during SARS-CoV-2 infection (149). SEVs carrying the SARS-CoV-2 spike protein were detectable in the circulation earlier than Abs to the SARS-CoV-2 spike protein in healthy individuals vaccinated with the mRNA BNT162b2 (Pfizer–BioNTech) vaccine (149). Mice immunized with the sEVs carrying the spike protein also developed Abs to the SARS-CoV-2 spike protein, indicating the immunogenicity of sEVs *in vivo* (**Figure 3**).

## MicroRNA’s in EVs and Their Role in Immune Response

Several studies have demonstrated that microRNAs (miRNAs) play a major role in the regulation of allograft cells function in response to injury following solid organ transplantation (150–152). A recent integrative omics analysis of kidney allograft biopsies demonstrated several miRNA associated with microvascular inflammation-related pathways, in particular miRNA such as miR-142-3p/150-5p/155-5p/222-3p/223-3p (150). Earlier studies from our lab, and others, in organ transplantation have shown miRNA such as miR142-3p/5p (AMR) (153), miR155 (T cell activation and fibrosis) (154), miR223 (inflammation and aggravation of renal dysfunction) (151), miR10a (acute rejection) (155), let-7c (inflammation, cell migration, and proliferation) (156) can predict allograft status (157).

EVs are enriched in noncoding RNAs, of which miRNAs play an important role and the mechanisms regulating selective miRNA packaging into EVs are being explored (158). Earlier studies demonstrated in response to endotoxin stimulation, macrophages secrete EVPs containing miR-155, which in turn promotes inflammatory responses to LPS *in vivo* (159, 160). These studies provide evidence that miRNA transfer between immune cells facilitate the regulation of inflammatory responses (161). Dendritic cell derived EVs have been demonstrated to regulate innate immunity by EV-mediated transfer of miRNAs among DCs contributes to enhance their mutual activation during inflammation (162, 163). DC derived EVs with CD86, a costimulatory molecule, activates T cells through direct or semi-direct pathway (3, 164). A recent study in murine models of renal allografts demonstrated highly immature DC–derived EVs containing miR-682, suppressed CD4+ T cells and promoted regulatory T cell development, whereas mature DC–derived EVs induced T cell immunity (165). Treg-derived EVs have also been demonstrated to induce a tolerogenic phenotype of DC by transfer of miRNAs (miR-150-5p and miR-142-3p*)* (166). T cells also release sEVs and other EVs after T cell receptor activation having a dual role (immune suppression or activation) depending on the miRNA content and release (167, 168).

## EVs in Attenuating Immune Responses

Recent studies have demonstrated that sEVs also play a role in the induction of tolerance after transplantation (169, 170). Donor-derived sEVs possess immunoregulatory molecules and also carry cell-derived antigens and donor HLA molecules. After infusion of donor monocyte-derived regulatory cells 7 days before transplantation, living liver transplant donors demonstrated transiently elevated levels of donor HLA, and immunoregulatory PD-L1, CD39 and CD73 molecules were detected in circulating small EVs, which can promote an immunosuppressed environment (171). Extracellular adenosine is a well-studied neurotransmitter, but it also exerts profound immune regulatory effects on the tolerogenic functions of DCs s by activity of CD73 and CD39 (172). PD-L1 and CD73 also can induce T cell anergy leading to tolerance (164, 173, 174). In an earlier study, circulating sEVs carrying PDL-1 and CD73 inhibited antitumor activities in patients with acute myeloid leukemia by delivering their suppressive cargos to immune recipient cells (175). Further, several reports have shown that allogeneic sEVs and T regulatory cell–derived sEVs can be used to promote tolerance to allografts (164, 176–178). A recent report demonstrated that T regulatory cell–derived IL-35-coated EVs can promote infectious tolerance (179).

## Conclusions and Future Perspectives

Several clinical and pre-clinical studies have demonstrated that immune responses to mismatched donor HLA and tissue-associated non-HLA TaAgs play an important role in the rejection of solid organ transplants; however, many questions remain unanswered. Pre- and post-transplant HLA-DSAs are now routinely monitored not only to select histocompatible donors but also to identify transplant recipients at risk for rejection so that appropriate treatment strategies can be instituted. Identification of sensitization to non-HLA tissue-associated Ags both before and after transplantation is likely to improve the outcome of the transplanted organ. This may also impact long-term survival of allografts by preventing or delaying onset of chronic rejection, which remains a major problem following organ transplantation. Currently, only a limited number of non-HLA tissue-associated Ags have been identified, and their role in rejection has been proposed. Certainly, other molecules and receptors that play a role in allograft rejection are yet to be discovered. For example, muscarinic receptor is a G protein-coupled receptor-like AT1R, distributed in the lungs, that has a distinct functional role in lung physiology. An earlier study demonstrated that high levels of Abs to muscarinic receptors in sera altered immune regulation in patients with chronic fatigue syndrome (180). It is clear from the recent literature that sEVs released from donor organs play an important role in inducing rejection, in particular chronic rejection. Donor-derived sEVs possess immunoregulatory molecules and also carry cell-derived antigens and donor HLA molecules. Apart from intercellular communication, the mechanisms by which sEVs regulate immune responses after transplant is yet to be explored. However, it is evident that circulating sEVs with tissue-associated antigens synergistically mediate alloimmune and autoimmune responses. Therefore, it is important to define the role of circulating sEVs and identify strategies to selectively control the role of donor organ–derived sEVs since recipient sEVs are critical to normal physiology. Studies are also required to confirm the potential use of sEVs as a biomarker of allograft rejection, especially to identify transplant recipients at risk for developing chronic rejection so that strategies can be developed for preventing and treating this form of rejection, which remains a major impediment to long-term function of the transplanted organ, especially lung allografts. Finally, with further investigation, sEVs carrying viral antigens can likely play an important role in the early identification of various viral and bacterial infections affecting organ transplant recipients since circulating sEVs contain viral and bacterial antigens.

## Author Contributions

Concept and design: TM; Manuscript Drafting, writing, and Figures: RR, SB, MR, and TM; Critical review and support: AS, NS, TF, and AB. All authors contributed to the article and approved the submitted version.

## Funding

This work was supported by NIH R21AI123034, NIH HL056643 and St. Joseph’s Foundation (TM).

## Conflict of Interest

The authors declare that the research was conducted in the absence of any commercial or financial relationships that could be construed as a potential conflict of interest.

## Publisher’s Note

All claims expressed in this article are solely those of the authors and do not necessarily represent those of their affiliated organizations, or those of the publisher, the editors and the reviewers. Any product that may be evaluated in this article, or claim that may be made by its manufacturer, is not guaranteed or endorsed by the publisher.
